# Single-Imaging Parasite-Quantification Microfluidic Device for Detection and Analysis of Schistosoma Eggs in Urine

**DOI:** 10.3390/mi17020270

**Published:** 2026-02-22

**Authors:** Heaven D. Chitemo, Vyacheslav R. Misko, Matthieu Briet, Jeffer Bhuko, Filip Legein, Humphrey D. Mazigo, Wim De Malsche

**Affiliations:** 1 Department of Biochemistry & Molecular Biology, School of Medicine, Catholic University of Health and Allied Sciences, Mwanza P.O. Box 1464, Tanzania; chitemoheaven57@gmail.com; 2 µFlow Group, Department of Bioengineering Sciences, Department of Chemical Engineering, Vrije Universiteit Brussel, 1050 Brussels, Belgium; matthieu.briet@vub.be (M.B.); filip.legein@vub.be (F.L.); 3 Department of Pathology, School of Medicine, Catholic University of Health and Allied Sciences, Mwanza P.O. Box 1464, Tanzania; jeffbhuko@outlook.com; 4 Department of Medical Parasitology, School of Medicine, Catholic University of Health and Allied Sciences, Mwanza P.O. Box 1464, Tanzania; humphreymazigo@gmail.com

**Keywords:** neglected tropical diseases, microfluidics, Single Image Parasite Quantification (SIMPAQ), urinary schistosomiasis

## Abstract

The accurate diagnosis of schistosomiasis for effective disease surveillance, treatment, and follow-up is crucial to attain the World Health Organization’s 2030 goal to eliminate schistosomiasis as a public health problem. The current diagnostic tools for urinary schistosomiasis, including the gold standard urine filtration test, have been reported to show low sensitivity in detecting low-intensity infections, which, when missed, act as reservoirs for infections—an evident gap in endemic areas where preventive chemotherapy reduces infection intensities. This study assessed the laboratory-based performance of the newly developed urinary Single Imaging Parasite Quantification chip for *Schistosoma haematobium* egg detection across different infection intensities. Two designs of the urinary chips were evaluated using polystyrene particles as a model for *Schistosoma haematobium* eggs, where the prototype design effectively captured the particles in the field of view with 96.00% to 100% efficiency. The second-generation chip, while eliminating the need for the air-drying step that was necessary in the operation of the prototype chip, similarly showed high capture efficiencies (95.20% to 96.00%). Overall, the prototype chip slightly outperformed the second-generation chip, and this difference was statistically significant (unpaired *t*-test, *p* = 0.0319). Testing of the prototype chip with spiked goat urine maintained high efficiencies of 99.33% to 100%. Similarly, both chip designs could trap real *Schistosoma haematobium* eggs in their fields of view, demonstrating their potential as diagnostic platforms that can contribute to improved diagnostics, disease surveillance, and monitoring.

## 1. Introduction

Schistosomiasis is a neglected tropical disease (NTD) targeted by the World Health Organization (WHO) for elimination as a public health problem by 2030 [1]. Six species of the genus Schistosoma can infect humans: *S. mansoni*, *S. japonicum*, *S. guineensis*, *S. intercalatum*, and *S. mekongi*, causing intestinal schistosomiasis, and *S. haematobium*, causing urogenital schistosomiasis [1]. Globally, schistosomiasis affects around 230 to 240 million people [2], with an estimated 151.38 million cases by 2021, with Africa accounting for 84.25% of these cases [3]. *S. haematobium* infects more than 112 million people worldwide, with around 436 million people at risk of being infected [4].

Schistosomiasis control strategies include provision of clean water, sanitation, and hygiene; mass drug administration (MDA) with praziquantel; and improving diagnostics for timely diagnosis, surveillance, and monitoring [1]. The WHO’s gold standard test for urinary schistosomiasis is the urine filtration test, which concentrates eggs on a polycarbonate membrane (12 µm to 25 µm pore sizes) for microscopic enumeration of the number of eggs per 10 mL of urine [5,6,7]. Although specific, its sensitivity is limited in low-intensity infections (<50 eggs/10 mL urine), leading to missed cases and underestimation of community prevalence, with downstream implications for MDA scheduling [1,8,9,10]. Repeated testing can improve sensitivity but is often impractical during large-scale screening, especially in resource-limited settings [6,9,11,12].

Hematuria, a common sign of urogenital schistosomiasis, can serve as a diagnostic indicator for urinary schistosomiasis [13], where gross hematuria can warrant treatment in high-intensity settings [8,13]. Microscopic hematuria detected by chemical reagent urinalysis dipsticks is rapid, cheap, and requires minimal expertise; however, it is challenged by variable sensitivity, especially in low and ultra-low intensities [8,14,15,16]; variable performance across brands; and lack of specificity, limiting its use as a standalone test [14,15,17]. Serological tests detecting schistosome antigens or antibodies can support diagnosis, but their utility is limited by false-negative results during early stages of infection, variable and unpredictable host immune response, cross-reactivity, persistence of antibodies from past infections, and lack of standardized antigens [10,18,19]. Antigen detection tests such as circulating cathodic antigen (CCA) and circulating anodic antigen (CAA) in blood or urine offer higher sensitivity; however, CCA shows reduced accuracy for *S. haematobium*, while CAA testing is still laboratory-dependent and costly [18,20,21,22]. Molecular-based assays like polymerase chain reaction (PCR) provide excellent sensitivity and specificity but require specialized equipment and trained personnel, limiting their field deployment [9,18].

To address these issues, the WHO Diagnostic Technical Advisory Group has outlined target product profiles (TPPs) to guide the development of ideal diagnostic tools [18]. Ideal tests should be highly sensitive, especially to low- and ultra-low-intensity infections; specific; point-of-care; portable; and functional in zero-infrastructure settings, allowing for testing in outdoor and resource-limited environments. They should require minimal sample preparation, low sample volumes, produce stable and easily interpretable quantitative results, minimize operator steps (≤5), rely on widely available materials, and be safe, robust, and suitable for use by minimally trained personnel [18].

Regarding the challenges associated with parasite egg detection and analysis, microfluidic devices may offer several advantages, such as portability, low sample and reagent use, reduced contamination risk, high sensitivity, and short analysis times, which are among the ideal TPPs outlined by WHO [18,23,24]. Microfluidic platforms, both passive, active, and hybrid operating devices, have been widely used in the manipulation, separation, and detection of biological materials like cells, bacteria, and parasites [23,24,25]. Passive devices include size-based filtration, deterministic lateral displacement (DLD), and inertial microfluidics. These rely on channel geometry and hydrodynamic forces without external energy input to trap or separate components [26]. Filtration-based devices have previously been applied in the detection of *S. haematobium* eggs [27], but their operation requires controlled flow conditions attained by the use of a syringe pump. Active operating devices like dielectrophoresis (DEP) and acoustofluidics enable highly selective particle manipulations, including live/dead discrimination of microorganisms [28], but generally depend on external power, integrated electrodes or transducers, and complex control electronics. Hybrid and three-dimensional (3D) microfluidic platforms combine active and passive strategies to improve throughput and analytical performance [29], yet they involve complex fabrication, strict operation requirements, and higher user training demands. While these approaches offer powerful particle control, their reliance on precise flow regulation, external fields, and sophisticated fabrication [25] may limit their suitability as field-based diagnostics for resource-limited settings.

In this paper, we present a new urinary Single Imaging Parasite Quantification (SIMPAQ) chip designed to enable trapping of *S. haematobium* eggs using a flow-based mechanism, in which variations in chamber depth enable passive egg retention as the sample flows through the device. This new device adopts key elements (like the detection chamber and the imaging technique) of microfluidics-based, Lab-on-a-Disk (LOD) SIMPAQ devices initially designed for the detection and imaging of intestinal helminth eggs in stool [30] and subsequently modified to enhance sensitivity [31,32,33], with feasibility demonstrated under field conditions [34]. Earlier approaches allowing parasite egg detection in urine, such as the size-based filtration devices [27], demonstrated proof-of-concept feasibility but were tested on a limited number of spiked eggs, restricting generalizability. In this study, we characterize the performance of the SIMPAQ urinary chip in a laboratory-based setting across a wide range of particle concentrations using polystyrene particles as a model for *S. haematobium* eggs and further assess the feasibility using preserved urine samples from infected individuals. This study is positioned as a proof-of-concept and analytical performance characterization of the SIMPAQ urinary chip.

## 2. Materials and Methods

### 2.1. Overview of Urinary SIMPAQ Chip

**Figure 1:**
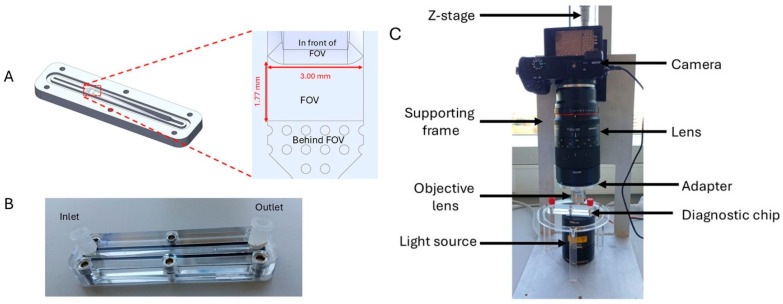
Urinary SIMPAQ chip and imaging setup. (**A**) Computer-aided design of the prototype urinary SIMPAQ chip, highlighting the field of view (FOV) and its dimensions. (**B**) An assembled prototype chip with Luer-lock adapters in place, ready for use. (**C**) A digital camera captures images of the FOV magnified by 10x objective lenses, illuminated by a halogen light source for improved visibility.

The chip is a reusable, point-of-care diagnostic device made from polymethyl methacrylate (PMMA; Eriks, Utrecht, The Netherlands) using computer numerical control milling (Datron Neo, DATRON AG, Ober-Ramstadt, Germany). It consists of two PMMA blocks, with the bottom block containing a 6.50 cm chamber with a 500 µL volume featuring an upward-sloping floor that tapers into a shallow field of view (FOV), which is 1.77 mm long, 3.00 mm wide, and 60–120 µm deep, designed to trap *S. haematobium* eggs (110–170 μm long and 40–70 μm wide) [31] in a monolayer. Behind the FOV, towards the outlet, a shallow (20 µm deep) pillar region allows continuous fluid flow toward the outlet (Figure 1A). The top block includes screw-fit holes and Luer-lock-compatible inlet and outlet ports (Figure 1B). Chamber depths were verified using a stylus profilometer (DektakXT, Bruker, Kontich, Belgium) before use.

### 2.2. Imaging Setup

The imaging setup consists of a Sony α6100 camera (Sony Group Corporation, Minato, Tokyo, Japan) fixed on a supporting frame. The camera is paired with a Samyang Macro lens (2.8/100 mm, Samyang Optics, Masan, Republic of Korea), which is attached to a 10× magnification objective lens through an adapter. The height of the lens from the chip can be controlled by rotating the knob of the Z-stage. To enhance image visibility, a halogen light source (quartz tungsten–halogen lamp, Thorlabs Inc., Newton, NJ, USA) is used to illuminate the devices (Figure 1C).

### 2.3. Model Particles for Assessment of the Urinary SIMPAQ Chip

Initial testing of the urinary chips was done using polystyrene particles (Microparticles GmbH, Berlin, Germany) as a model of *S. haematobium* eggs, which measure 110–170 µm in length and 40–70 µm in width [35]. Blue polystyrene particles (diameter: 123 ± 4.10 µm) and red polystyrene particles (diameter: 60 ± 1.20 μm), both with a density of 1.05 g/mL, were selected to approximate *S. haematobium* eggs’ length and width, respectively. In this way, the particles enable modeling of the dispersion of eggs in size and their various orientations inside the device. Particle suspensions at a concentration of five particles/μL were prepared in deionized water (Millipore Synergy UV, Spectralab Scientific Inc., Markham, ON, Canada) and stored at room temperature. To simulate different infection intensities, 5 (ultra-low), 10 (very low), 25 (low), 50, and 100 (high intensity) particles were suspended in 500 µL of water and injected into water-prefilled chips.

### 2.4. Sample Analysis for Urinary SIMPAQ Chip

The working protocol for the prototype urinary SIMPAQ chip involves a one-syringe system where sample loading into the chip was performed manually using a standard disposable syringe. Typically, 500 µL of the particle suspension was injected into the chip’s inlet over approximately 20–30 s, corresponding to an estimated flow rate of ~1.0 to 1.5 mL/min, followed by a 5 mL water flush to ensure that all particles were delivered to the FOV. An air pulse is then given to remove excess liquid and allow particles to remain stationary for imaging. During these steps, the chip is held in a vertical orientation to prevent particles from sinking and settling at the bottom of the chamber instead of the FOV. This manual operation was chosen to reflect realistic field-use conditions where sophisticated materials like syringe pumps are unavailable. For each particle load (5, 10, 25, 50, and 100 particles), five repeated experiments were performed.

The FOV capture efficiency was the primary quantitative performance metric of the chip. For each particle load, it was calculated as

(Np_FOV)/(Np_inj) × 100%

where Np_inj and Np_FOV are the numbers of injected particles and those in the FOV, respectively.

## 3. Results

The results presented in this study focus on proof-of-concept validation, analytical performance characterization, and feasibility using both polystyrene particles and preserved infected urine samples. This work does not aim to establish diagnostic accuracy or direct equivalence with standard urine filtration methods at this stage.

### 3.1. Urinary SIMPAQ Chip Accurately Detects Ultra-Low to High Polystyrene Particle Loads

**Figure 2:**
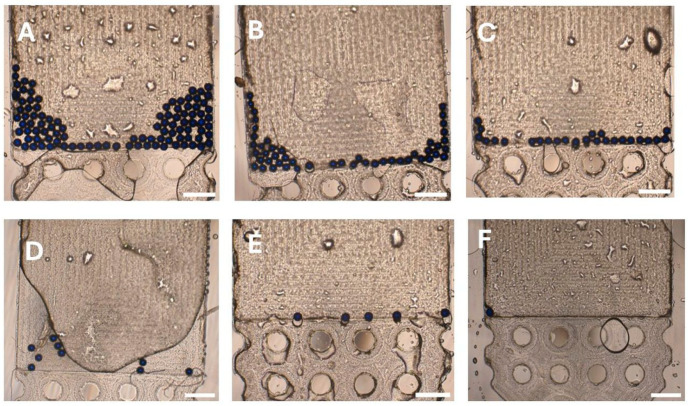
Field-of-view (FOV) particle capture in the prototype urinary SIMPAQ chip. (**A**) N = 100 particles, mean capture efficiency 98.60%. (**B**) N = 50 particles, 98.80%. (**C**) N = 25 particles, 100%. (**D**) N = 10 particles, 96.00%. (**E**) N = 5 particles, 96.00%. (**F**) Consistent capture of a single particle in the FOV across five repeated experiments. Scale bars = 500 µm.

To evaluate the performance and efficiency of the prototype urinary SIMPAQ chip, different particle concentrations were tested using the proposed protocol. The chip’s efficiency was then recorded as mean (±standard deviation) FOV capture efficiency across five repeated experiments for each particle load. The high-intensity particle loads, 100 and 50 particles, had mean FOV capture efficiencies of 98.60% (±0.55) and 98.80% (±1.10), respectively (Table 1; Figure 2A,B). Similarly, the low-intensity particle loads with 25 particles had a mean FOV capture efficiency of 100%, while 10 and 5 particles had mean FOV capture efficiencies of 96.00% (±5.48) and 96.00% (±8.94), respectively (Table 1; Figure 2C–E). Although the five-particle load group had the greatest variability (standard deviation = 8.94), four out of the five repeated experiments achieved 100% FOV capture efficiency. To further examine the strength of the chip at ultra-low-infection intensities, a single-particle load was analyzed, where it was successfully delivered to the FOV in all five attempts (Figure 2F).

Air drying of the chamber (i.e., removing excess fluid while keeping the particles wet) prevents fluid backflow when the inlet syringe is detached for imaging. This procedure maintains the particles’ position within the chamber, allowing easy manipulation and examination of the whole chip under the imaging setup with no disruption of the particles’ position.

### 3.2. The Prototype Chip Consistently Achieves High Capture Efficiency at Higher Particle Loads

To assess the upper functional limit of the prototype chip, the mean FOV capture efficiency was determined at higher particle loads. The World Health Organization defines high-intensity *S. haematobium* infections as those with 50 or more eggs per 10 mL of urine [36]. To test the device’s upper limit, hypothetical subcategories of high-intensity infection simulations were introduced and tested: high-heavy (170 particles), very high-heavy (350 particles), and extreme-heavy (500 particles). Each category was then tested in three repeated experiments to determine the mean FOV capture efficiency.

**Figure 3:**
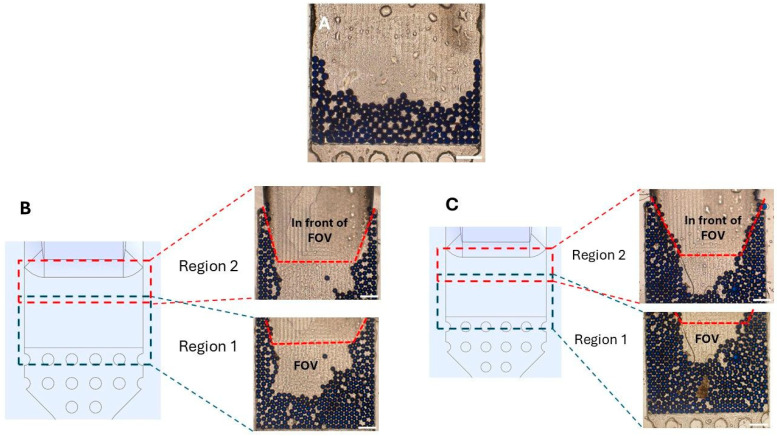
The prototype urinary SIMPAQ chip’s field-of-view (FOV) particle capture and behavior in high-intensity loads. (**A**) N = 170 particles—mean capture efficiency 99.22%. (**B**) N = 350 particles, 98.00%. (**C**) N = 500 particles, 94.20%. Higher particle loads (350 and 500) resulted in FOV saturation, where full visualization requires overlapping images (**B**,**C**). Scale bars = 500 µm.

The chip maintained a high mean (±standard deviation) FOV capture efficiency even in this category of tests (Table 2). At 170 particles, the mean FOV capture efficiency was 99.22% (±1.36), where all particles in the chip were in the FOV in all experiments (Figure 3A). At 350 particles, the mean FOV capture efficiency slightly declined to 98.00% (±1.14), with a mean of four particles observed close to, but just outside, the FOV (Figure 3B). This was more pronounced at 500 particles, where the FOV was almost fully crowded with particles. The capture efficiency reduced further to 94.20% (±1.00) with a mean of seven particles just outside the FOV (Figure 3C).

A practical observation during testing of these high particle loads, especially 350 and 500 particles, was the difficulty in capturing all particles in the FOV within a single camera shot. Even though particles were in the FOV or nearby, their distribution extended beyond the camera boundaries. As a result, obtaining a complete visual required taking at least two overlapping images (Figure 3B,C).

### 3.3. Second-Generation Urinary SIMPAQ Chip Offers High-Field-of-View Capture Efficiency Without the Need for Air-Drying

**Figure 4:**
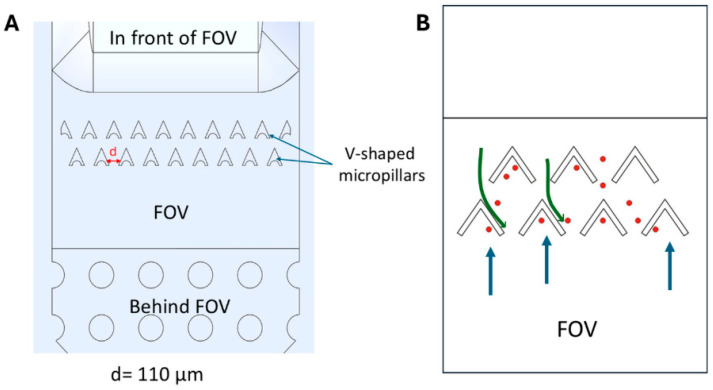
Design and functional illustration of the second-generation urinary SIMPAQ chip. (**A**) Computer-aided design of the second-generation urinary SIMPAQ chip with V-shaped micropillars in the field of view (FOV) spaced 110 μm apart. Dimensions of the FOV are the same as the prototype design. (**B**) Schematic illustration of the operational principle of the chip: during forward fluid flow (green arrows), polystyrene particles (red dots) pass through the micropillars to accumulate in the FOV. In reverse flow (blue arrows), the micropillars interfere with the reverse movement of particles, preventing their loss and eliminating the need for a drying step.

The WHO Target Product Profile highlights that an ideal diagnostic test should have minimal operator steps (≤5) to reduce result variations and facilitate automation when needed [18]. Air-drying in the prototype chip introduces an additional step, where the user must switch the fluid delivery syringe to another one filled with air to dry the chamber. The additional syringe manipulations may not be ideal, especially when using real urine samples, as they may complicate efforts to automate the diagnostic process and may result in contamination. To address this, a second generation of the urinary SIMPAQ chip was designed. While maintaining similar dimensions as the prototype chip, the FOV of the second-generation chip incorporates V-shaped micropillars spaced *d* = 110 μm apart (Figure 4). These structures allow entry of particles into the FOV during forward fluid flow while interfering with particle backflow caused by fluid suction during syringe manipulations, therefore eliminating the need for drying. The gap between the V-shaped micropillars (*d* = 110 μm) was selected to allow the passage of *S. haematobium* eggs when oriented along the direction of flow. The V-shape of the micropillars facilitates such egg orientation when flowing towards the FOV (“easy” direction) while substantially reducing the probability of reverse passage during backflow (“hard” direction). During preliminary testing, uniformly sized blue polystyrene particles with diameters comparable to upper egg dimensions (diameter: 123 ± 4.10 µm) exhibited entry resistance at the micropillar array. Therefore, to evaluate the intended trapping performance, experiments with the second-generation urinary SIMPAQ chip used red polystyrene particles (diameter: 60 ± 1.20 μm) that fit into the inter-pillar gap. Importantly, unlike uniform spherical beads, *S. haematobium* eggs are asymmetric and non-spherical, which enables them to traverse the micropillar array under forward flow. The mean FOV capture efficiency was similarly determined across the standard particle loads (5, 10, 25, 50, and 100 particles) in five repeated experiments using red polystyrene particles.

**Figure 5:**
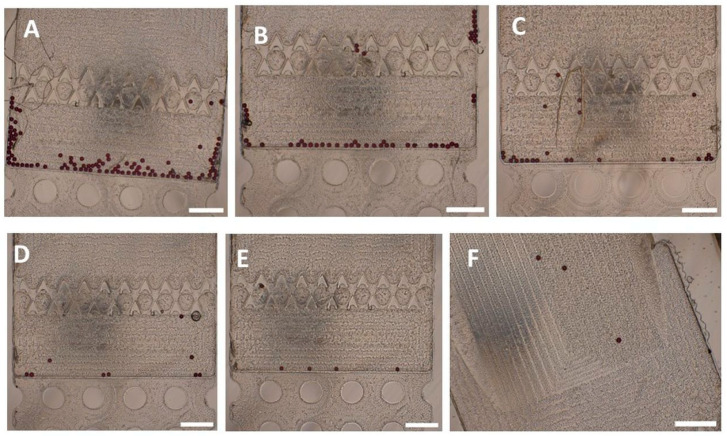
Field-of-view (FOV) particle capture in the second-generation urinary SIMPAQ chip. Second-generation chips have micropillars in the FOV that prevent particle backflow, eliminating the air-drying step. Five repeated experiments with (**A**) N = 100 particles—mean capture efficiency 95.80%, (**B**) N = 50 particles, 95.20%, (**C**) N = 25 particles, 96.00%, (**D**) N = 10 particles, 92.00%, and (**E**) 5 particles, 96.00%. (**F**) Occasionally, 1–3 particles escape the FOV when the chip is turned horizontally for imaging. Scale bars = 500 µm.

The mean (±SD) FOV capture efficiencies observed for the second-generation chip were: 95.80% (±2.77) for 100 particles, 95.20% (±2.28) for 50 particles, 96.00% (±2.83) for 25 particles, 92.00% (±8.37) for 10 particles, and 96.00% (±8.94) for 5 particles (Table 3; Figure 5A–E).

A practical observation with the second-generation chips was that, despite the presence of the micropillars, a few particles could still escape the FOV. To minimize this, all experiments using this chip were done using one syringe connected to the inlet while the chip was held in a vertical orientation. Additionally, after sample introduction into the chip and a fluid flush, the outlet port was sealed with a Luer-lock cap before removing the inlet syringe; the inlet port was then immediately sealed, also with a Luer-lock cap, while keeping the chip vertical. Still, 1–3 particles managed to escape when the chip was turned horizontally for imaging (Figure 5F), thus accounting for the lower observed FOV capture efficiencies. This occurs because during syringe detachment and sealing, loss of even a minimal amount of fluid can create a vacuum that is sufficient to cause a fluid shift when the chip is turned horizontally for imaging. Therefore, care is needed to prevent particle loss after successfully collecting them in the FOV.

The mean FOV capture efficiencies in the second-generation chip were slightly lower compared to those obtained in the prototype design using the one-syringe air-drying protocol. The differences in capture efficiency were statistically significant at 50 (unpaired *t*-test, *p* value = 0.0202, indicating statistical significance) and 25 particle loads (*p* value = 0.0341). In the other particle loads (5, 10, and 100 particles), no statistically significant difference was found (*p* values = 1, 0.4012, and 0.0864, respectively). The overall mean capture efficiency across all different particle loads for the prototype chip was 97.88%, which was significantly higher than that for the second-generation chip at 94.96% (*p* value= 0.0319) (Table 4), thus, showing that the prototype chip design demonstrates a slightly higher capture efficiency than the second-generation chip design.

### 3.4. SIMPAQ Chip Performance Using Spiked Real Urine and Real Infected Urine

**Figure 6:**
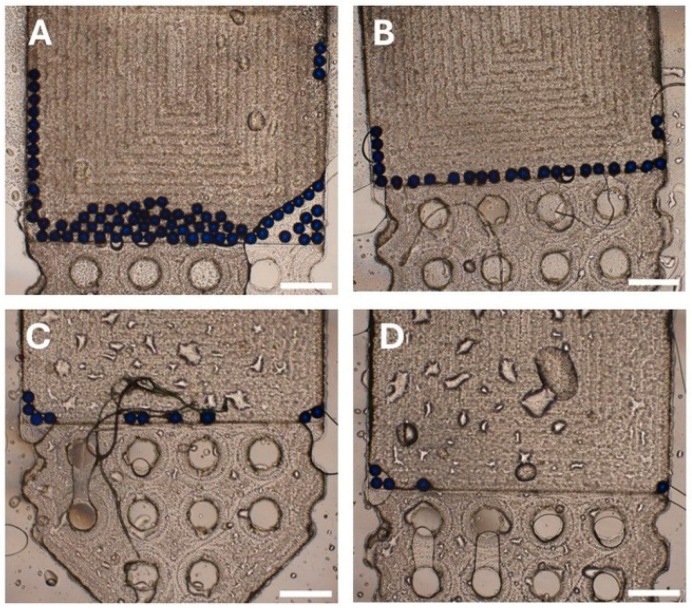
Field-of-view (FOV) capture efficiency of the prototype chip in real urine samples. The one-syringe air-drying protocol was used to determine FOV capture efficiency after spiking goat urine with polystyrene particles. (**A**) N = 75 particles—mean capture efficiency 99.33%, (**B**) N = 25 particles, 100%, (**C**) N = 10 particles, 100%, and (**D**) N = 5 particles, 100%. Scale bars = 500 μm.

To assess the performance of the urinary SIMPAQ chip using real urine samples, the mean FOV capture efficiency was determined using the prototype chip with the one-syringe air-drying protocol. In total, 500 μL of goat urine was spiked with blue polystyrene particles (5, 10, 25, and 75 particles), which were then injected into the chip, followed by a water flush. Afterwards, the chip was air-dried and examined using the imaging setup. Each particle load was tested twice. The mean (±SD) FOV capture efficiencies observed were 99.33% (±0.94) for 75 particles and 100% for 25, 10, and 5 particles (Table 5; Figure 6). Suspension of the particles in urine did not cause any operational challenges or noticeable changes in particle behavior.

**Figure 7:**
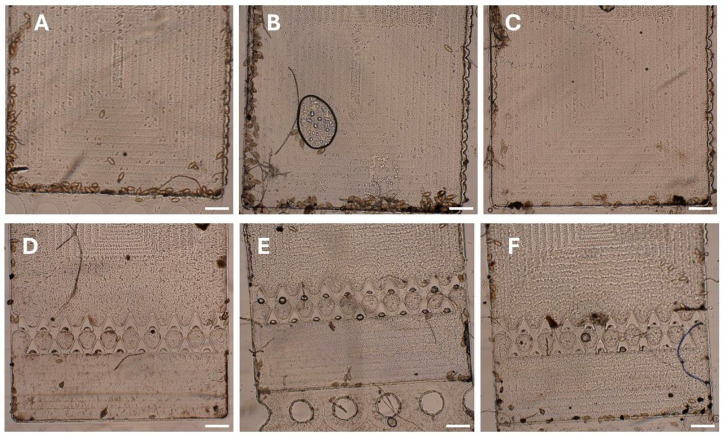
Field-of-view (FOV) Capture of *S. haematobium* eggs from infected human urine samples. (**A**–**C**) Schistosoma eggs captured at the FOV of the prototype urinary SIMPAQ chip. (**D**–**F**) Egg capture at the FOV of the second-generation chip. Scale bars = 500 μm.

Real infected human samples were also tested using both the prototype and second-generation chips to assess the chips’ ability to capture real *S. haematobium* eggs and assess the need for any sample pretreatment. *S. haematobium*-infected human samples were obtained from preserved stock from a disease monitoring study in primary schools in Meatu District in Simiyu Region, Tanzania. The urinary SIMPAQ chip was shown to trap real *S. haematobium* eggs at the FOV as identified by their characteristic terminal spine (Figure 7). An important operational consideration noted is that the direct loading of fresh urine into the chip led to rapid egg hatching under the halogen light illumination, releasing motile miracidia that hindered proper chip examination due to the rapid movement of miracidia that occasionally escaped the FOV. To mitigate this, adding a few drops (3–5) of 1:5 diluted Lugol’s iodine in deionized water to the urine sample effectively prevented hatching and allowed sufficient time for examination and image acquisition. This simple and low-cost pretreatment step is a practical modification that enhances the usability of the chips.

## 4. Discussion

Microfluidics-based platforms such as SIMPAQ represent promising diagnostic alternatives for helminth infections [30]. This study aimed to evaluate the newly developed flow-based urinary SIMPAQ chips for the detection of *S. haematobium* eggs in urine. The device was tested in laboratory-based settings using a wide concentration range of polystyrene particles as a model for *S. haematobium* eggs to assess performance and operational behavior. This was followed by feasibility testing using preserved infected urine samples. Although FOV capture efficiency was the primary quantitative performance metric evaluated, our experimental findings also provide qualitative insight into several observations associated with the use of our platform.

The urinary SIMPAQ chip demonstrated stable performance across a wide range of particle loads, from single particle detection to highly saturated conditions (500 particles/500 µL water), without observable clogging or flow interruption; a notable feature given the susceptibility of passive microfluidic systems, particularly filtration-based devices, to clogging when processing debris-rich biological samples [27,37]. The prototype chip, operated using a one-syringe, temporary air-drying protocol, demonstrated consistently high mean FOV capture efficiencies ranging from 96% to 100%, including the ability to capture a single particle in the FOV. This is critical, as low-intensity *S. haematobium* infections are often missed by current routine tests [8]. The ability of the SIMPAQ chip to reliably detect low particle loads highlights its potential as a diagnostic, surveillance, and follow-up platform pending further field validation in ultra-low- and low-intensity infection settings where such untreated infections can sustain transmission [16,38].

At higher simulated infection intensities, the SIMPAQ chip maintained high capture efficiencies (99.22% for 170 particles, 98.00% for 350 particles, and 94.20% for 500 particles). This represents a promising alternative to conventional urine filtration methods, particularly for analytical applications where high egg retention is important, as egg loss at higher egg burdens has been reported in routine filtration-based approaches [37]. Although such losses do not compromise clinical decision-making, since the presence of any eggs warrants treatment with praziquantel, these losses have significant implications in research and disease surveillance settings, such as monitoring treatment efficacy or studying the effectiveness of alternative dosing regimens [38].

Manually operated passive microfluidic systems exist for sorting different biological particles. Song et al. [39] described a continuous flow hydrophoretic syringe-driven device capable of size-based separation of cells, where slanted ridge structures induce size-dependent cell fractionation. Similarly, portable, hand-powered particle sorting devices exist where flow in a groove-based channel drives size-based separation of particles suspended in water and cells in human blood [40]. These platforms demonstrate the feasibility of such systems for sample sorting; however, they are designed for continuous separation rather than immobilization and image-based quantification and are sensitive to unstable and high flow rates [39,40]. In contrast, the SIMPAQ chip adopts a depth-based geometric trapping strategy that immobilizes particles within a defined field of view, enabling direct single-image enumeration rather than outlet-based sorting. Under manual syringe-driven flow, the chip operated at approximately 1.0 to 1.5 mL/min without observable reduction in trapping efficiency within the tested range. Other size-based filtration devices offer proof-of-concept feasibility for schistosomiasis diagnosis, but these are typically evaluated over narrow egg count ranges and require controlled flow conditions, such as the use of syringe pumps, to maintain performance [27]. Deterministic lateral displacement and inertial microfluidic approaches can achieve continuous flow separation and high throughput but similarly depend on stable flow rates to function reliably [26]. Such requirements may limit large-scale field deployment, especially in resource-limited settings where schistosomiasis is endemic. Electric field-based manipulations like DEP provide highly selective particle control [28] but rely on external power sources, integrated electrodes, and control electronics, making them complex and limiting their use in resource-limited settings. Similarly, hybrid microfluidic platforms involve costly and high-infrastructure manufacturing and operation, and require extensive user training for operation [29]. In contrast, the SIMPAQ chip uses a purely manual flow-based trapping strategy without the need for precise flow control or external power, prioritizing operational simplicity, tolerance to manual flow variation, and minimal infrastructure requirements. This design and operational simplicity align with the WHO TPP priorities in resource-limited, field-based settings [18].

Although the SIMPAQ chips are designed for use as point-of-care diagnostic devices, the current operation relies on several manual steps, such as syringe handling, controlled chip orientation, and imaging using an external imaging setup. Suitable point-of-care devices are preferably portable and automated, with several automated microfluidic platforms available in the literature where sample introduction, analysis, and sensing are fully automated; they are limited by technical requirements requiring complex instruments [41]. Smartphone-based mobile health platforms also exist that integrate microfluidic detection, imaging, and computational analysis [42]. Similarly, these require advanced hardware, electronics, and software. The current SIMPAQ design allows immediate transfer of acquired images to a computer or smartphone using a USB connection. Future adaptations may include smartphone-based imaging, digitalized egg identification and enumeration, and enhancing point-of-care accessibility without compromising usability in field settings.

The air-drying protocol has an additional step requiring multiple syringe changes, which may not be ideal (although this procedure of removing excess fluid perfectly stabilizes the particle/egg positions collected in the FOV). As an alternative, a second-generation urinary SIMPAQ chip was developed to eliminate the air-drying step while preventing backflow by adding V-shaped micropillars in the FOV. This design had comparable (although slightly lower) overall capture efficiency (95.00%) compared to the prototype with the air-drying protocol (97.88%), a statistically significant difference (unpaired *t*-test, *p* value = 0.0319). Extra care should be taken when handling the second-generation chip because minor fluid losses during syringe detachment can result in bubbles that may introduce significant particle shifts during the horizontal placement of the chip for imaging. Such disruptions can cause the particles that were initially trapped in the FOV to potentially surpass the micropillars. This degree of care is not needed with the air-drying protocol. Such sensitivity of the system to minor deviations may pose challenges during field use, especially with less experienced users or when the test is performed in unfavorable environments like outdoor settings. Field-based diagnostic techniques must be compatible with environmental fluctuations while maintaining stable results even when used by the lowest cadres [18]. In this regard, considering simplicity and robustness, the prototype chip with the air-drying protocol outperforms the second-generation design.

Compatibility with urine samples was demonstrated by testing goat urine spiked with polystyrene particles, where near-perfect capture efficiencies (99.33% to 100%) were observed across different particle loads tested. This is a promising finding because it shows that despite the complex and biological nature of urine [43,44], which may affect microfluidic performance, the chips and the protocol still perform well. Feasibility testing of the chip with *S. haematobium*-infected human urine further demonstrated its ability to capture real eggs at the FOV.

Direct loading of fresh urine led to rapid egg hatching under the halogen light used for illumination to enhance visibility in the imaging setup, releasing miracidia, which interfered with stable visualization. A simple pretreatment with a few drops of iodine effectively prevented this and enabled clear, prolonged imaging. This minor adjustment improves the chip’s operation without adding procedural complexity. Although iodine is widely used in parasitology to enhance visualization of helminth eggs and immobilize motile stages [45], its specific effect on preventing hatching is not well characterized but is likely due to membrane fixation inhibiting the processes required for miracidia emergence.

Despite these promising findings, several limitations should be acknowledged. All experiments in this study were performed by a single researcher under controlled laboratory conditions, and user variability and operational robustness across different operators and sample introduction speeds were not systematically evaluated. In addition, performance assessment focused primarily on FOV trapping efficiency, with feasibility demonstrated using preserved infected urine samples. Direct quantitative comparisons with routine diagnostic tests, as well as sensitivity assessments in field conditions, were beyond the scope of the present study. These aspects will be addressed in planned, large-scale, field-based studies.

## 5. Conclusions and Perspectives

To attain health and well-being for all, collaborative efforts are necessary to cope with neglected tropical diseases, involving the improvement of helminth diagnosis using science and technology innovations. The urinary SIMPAQ chip is a microfluidic platform designed to enable egg detection and quantification of *S. haematobium* eggs in urine. The development and operation of the chip are aimed at addressing the World Health Organization’s target product profiles, relying on flow-based trapping of eggs in the imaging zone. This simple technique demonstrated efficient (96.00–100%) and consistent trapping of polystyrene particles as a model for *S. haematobium* eggs suspended in water across a wide range of particle sizes. Similarly, we showed that the current chip design is compatible with urine as a sample, as it achieved high FOV capture efficiency (99.33% to 100%) of polystyrene particles spiked in urine. Similarly, compatibility testing with real infected urine showed that the chip can effectively trap real *S. haematobium* eggs.

This study demonstrates the feasibility and analytical performance of the SIMPAQ chip for *S. haematobium* egg capture. The present work focuses on validating the design concept and trapping efficiency. Moving forward, a subsequent phase will involve large-scale field comparative studies incorporating standard urine filtration to assess robustness, user variability, and diagnostic performance in real field conditions.

## Figures and Tables

**Table 1:** Prototype urinary SIMPAQ chip’s field-of-view (FOV) capture efficiency using blue polystyrene particles.

Particles Injected	Mean Number of Particles in FOV *	Mean FOV Capture Efficiency (%)	Standard Deviation (SD)
100	99	98.60	0.55
50	49	98.80	1.10
25	25	100	0
10	10	96.00	5.48
5	5	96.00	8.94

* Mean number of particles in FOV across five repeated experiments, rounded up to whole numbers.

**Table 2:** The prototype urinary SIMPAQ chip’s field-of-view (FOV) capture efficiency at higher polystyrene particle loads.

Particles Injected	Mean Number of Particles in FOV *	Mean Capture Efficiency (%)	Standard Deviation (SD)	Particles near FOV
170	169	99.22	1.36	0
350	343	98.00	1.14	4
500	471	94.20	1.00	7

* Mean number of particles in FOV across three repeated experiments, rounded up to whole numbers.

**Table 3:** Field-of-view (FOV) capture efficiency in the second-generation urinary SIMPAQ chip using red polystyrene particles.

Particles Injected	Mean Number of Particles in FOV *	Mean FOV Capture Efficiency (%)	Standard Deviation (SD)
100	96	95.80	2.77
50	48	95.20	2.28
25	24	96.00	2.83
10	9	92.00	8.37
5	5	96.00	8.94

* Mean number of particles in FOV across five repeated experiments, rounded up to whole numbers.

**Table 4:** Comparison of the field-of-view (FOV) capture efficiency of the prototype and the second-generation SIMPAQ urinary schistosomiasis diagnostic chip design.

Particles Injected	Mean FOV Capture Efficiency (%)		*p* Value
	Prototype Chip Design	Second-Generation Design	
100	98.60	95.80	0.0864
50	98.80	95.20	0.0202 *
25	100	96.00	0.0341 *
10	96.00	92.00	0.4012
5	96.00	96.00	1.0000
Overall Mean Capture (%)	97.88	95.00	0.0319 *

* p values *<* 0.05 (bold) show a statistically significant difference.

**Table 5:** Field-of-view (FOV) capture efficiency in urine samples spiked with polystyrene particles using the prototype chip.

Particles Injected	Mean Number of Particles in FOV *	Mean FOV Capture Efficiency (%)	Standard Deviation (SD)
75	74.5	99.33	0.94
25	25	100	0
10	10	100	0
5	5	100	0

* Mean number of particles in FOV across two repeated experiments, rounded up to whole numbers.

## Data Availability

The original contributions presented in this study are included in the article. Further inquiries can be directed to the corresponding author.
